# Mito‐nuclear discordance across a recent contact zone for California voles

**DOI:** 10.1002/ece3.4129

**Published:** 2018-05-24

**Authors:** Dana Lin, Ke Bi, Christopher J. Conroy, Eileen A. Lacey, Joshua G. Schraiber, Rauri C. K. Bowie

**Affiliations:** ^1^ Museum of Vertebrate Zoology University of California, Berkeley Berkeley California; ^2^ Department of Integrative Biology University of California, Berkeley Berkeley California; ^3^ Computational Genomics Resource Laboratory California Institute for Quantitative Biosciences University of California, Berkeley Berkeley California; ^4^ Department of Biology Center for Computational Genetics and Genomics Temple University Philadelphia Pennsylvania; ^5^ Institute for Genomics and Evolutionary Medicine Temple University Philadelphia Pennsylvania

**Keywords:** contact zone, genomic markers, introgression, *Microtus*

## Abstract

To examine the processes that maintain genetic diversity among closely related taxa, we investigated the dynamics of introgression across a contact zone between two lineages of California voles (*Microtus californicus*). We tested the prediction that introgression of nuclear loci would be greater than that for mitochondrial loci, assuming ongoing gene flow across the contact zone. We also predicted that genomic markers would show a mosaic pattern of differentiation across this zone, consistent with genomes that are semi‐permeable. Using mitochondrial cytochrome *b* sequences and genome‐wide loci developed via ddRAD‐seq, we analyzed genetic variation for 10 vole populations distributed along the central California coast; this transect included populations from within the distributions of both parental lineages as well as the putative contact zone. Our analyses revealed that (1) the two lineages examined are relatively young, having diverged ca. 8.5–54 kya, (2) voles from the contact zone in Santa Barbara County did not include F1 or early generation backcrossed individuals, and (3) there appeared to be little to no recurrent gene flow across the contact zone. Introgression patterns for mitochondrial and nuclear markers were not concordant; only mitochondrial markers revealed evidence of introgression, putatively due to historical hybridization. These differences in genetic signatures are intriguing given that the contact zone occurs in a region of continuous vole habitat, with no evidence of past or present physical barriers. Future studies that examine specific isolating mechanisms, such as microhabitat use and mate choice, will facilitate our understanding of how genetic boundaries are maintained in this system.

## 1 INTRODUCTION

Understanding the processes that generate and maintain genetic differences among organisms is central to understanding the evolution of biodiversity (Seehausen et al., 2014; Vellend, 2005). Contact zones—areas where genetically distinct lineages meet and potentially interbreed—provide important natural laboratories for exploring the factors that promote or impede exchange of genetic material (Harrison, 1993; Hewitt, 1988). Characterizing patterns of genetic variation across a contact zone can yield important insights into these factors, including the potential roles of selective versus other processes such as neutral diffusion (i.e., dispersal over time; Endler, 1977) in determining the extent of introgression among lineages (Barton & Hewitt, 1985; Gay, Crochet, Bell, & Lenormand, 2008; Moore, 1977). More specifically, analyses of how genetic diversity is distributed across a contact zone can be used to infer whether selection acts for or against hybrid individuals (Moore, 1977) as well as to assess the degree of tension between selection and dispersal in maintaining genetic barriers between lineages (Endler, 1977; Jiggins & Mallet, 2000; Slatkin, 1973). As a result, analyses of the widths and shapes of clines across a contact zone are fundamental to efforts to understand the processes contributing to phyletic diversity.

Patterns of genetic diversity across a contact zone may differ among portions of the genome, in particular between mitochondrial and nuclear loci. Such discordance between mitochondrial and nuclear markers is not uncommon (Toews & Brelsford, 2012) and may reflect their distinct modes of inheritance and differences in effective population size (Ballard & Whitlock, 2004). The nature and extent of these differences can also provide insights into underlying processes, including the potentially significant roles of demography and historical events in shaping current patterns of genetic diversity (Edwards, Potter, Schmitt, Bragg, & Moritz, 2016; Good et al., 2008; Hey & Pinho, 2012; Hird & Sullivan, 2009; Oswald, Overcast, Mauck, Andersen, & Smith, 2017; Singhal & Moritz, 2013). As a result, generating data from both mitochondrial and nuclear genomes may be critical to understanding the maintenance of genetic differences across contact zones, particularly for recently diverged taxa or for contact zones not associated with conspicuous geographic features (Burton & Barreto, 2012; Coyne & Orr, 2004c; Hadid et al., 2013; Harrison & Burton, 2006; Toews & Brelsford, 2012).

**Figure 1:**
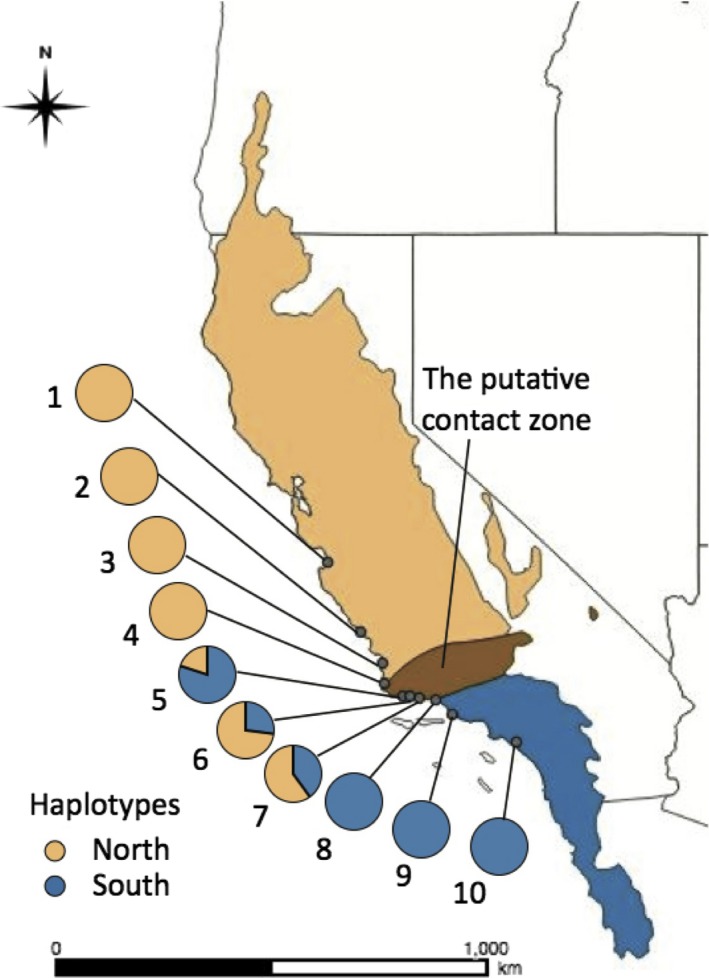
Distribution of northern (orange) and southern (blue) cyt‐*b* haplotypes along coastal California. Data are from cyt‐*b* sequences from 10 to 15 individuals per locality for 10 localities spanning the putative contact zone (brown) between lineages identified by Conroy and Neuwald (2008). Additional information for each sampling locality is given in Table 1; numbers assigned to sampling localities correspond to those in the table

The California vole (*Microtus californicus*) is a small, grassland rodent that occurs along the entire length of California. This species is divided into northern and southern clades that come into contact in Santa Barbara County (Figure 1) (Conroy & Neuwald, 2008). The genus *Microtus* appears to have undergone a relatively recent radiation in North America (ca. 1.3 mya; Conroy & Cook, 2000), implying a recent origin for these two lineages of *M. californicus*. Analyses of mitochondrial cytochrome *b* (cyt‐*b*) sequences indicate that these lineages are ca. 4.6% divergent (Conroy & Neuwald, 2008), a value that is consistent with species‐level cyt‐*b* sequence differences in other mammals (Matocq, 2002; Tobe, Kitchener, & Linacre, 2010). Comparisons of sequences from the cyt‐*b* locus and a nuclear intron (AP5) have revealed a discordance between these markers (Conroy & Neuwald, 2008), the spatial structure of which suggests that either (1) gene flow between lineages is sex biased or (2) some hybrid genotypes experience reduced fitness. Laboratory crosses indicate that F1 male hybrids are sterile (Gill, 1984), providing evidence that the genomes of the two parental lineages may be incompatible. Collectively, these lines of evidence suggest the presence of a partial reproductive barrier between northern and southern lineages of *M. californicus*, providing an important opportunity to examine the mechanisms maintaining genetic differences among lineages of conspecifics.

To identify the processes contributing to the maintenance of distinct genetic lineages of *M. californicus* in Santa Barbara County, California, we used both mitochondrial and genome‐wide nuclear markers to test two sets of predictions regarding the nature of the putative contact zone. First, if this zone reflects speciation with gene flow (Feder, Egan, & Nosil, 2012; Nosil, 2008) associated with Bateson‐Dobzhansky‐Muller incompatibilities (Coyne & Orr, 2004c; Haldane, 1922), then multiple generations of backcrossing of hybrids should be evident and genomic loci should show a mosaic pattern of differentiation across the contact zone consistent with semi‐permeability of the diverging genomes; collectively, these features should result in a gradual transition between genomes (i.e., unimodal contact zone; Jiggins & Mallet, 2000). In contrast, if the contact zone represents divergence among lineages that is maintained by environmental factors (e.g., climate or habitat‐specific adaptations) (Coyne & Orr, 2004b; Nosil, Harmon, & Seehausen, 2009) or sexual selection (Coyne & Orr, 2004a, 2004d), then a sharp cline in genetic variation (i.e., bimodal hybrid zone; Jiggins & Mallet, 2000) is expected, with introgression limited to early generation hybrids (e.g., F1, Shurtliff, Murphy, & Matocq, 2014). To distinguish between these scenarios, we characterized patterns of introgression across the contact zone and then evaluated the ability of different selective and demographic (e.g., migration vs. incomplete lineage sorting; Charlesworth, Bartolome, & Noel, 2005) mechanisms to account for these patterns. In addition to providing insights into the maintenance of genetic differences between northern and southern lineages of California voles, our analyses generate new insights regarding the processes operating in contact zones between closely related, presumably recently diverged lineages that are not associated with conspicuous geographic or environmental barriers.

## 2 MATERIALS AND METHODS

### 2.1 Field collection of samples

Collection of tissue samples for *M. californicus* was conducted along a transect in California that extended from Monterey Bay (Monterey County) in the north to Irvine (Orange County) in the south. This transect consisted of 10 collecting localities that included the northern and southern lineages of this species as well as populations from the putative contact zone in Santa Barbara County (Conroy & Gupta, 2011; Conroy & Neuwald, 2008; Table 1 and Figure 1). Ten to 15 individuals were collected from each locality. Additional information regarding sample collection is provided in the Supplementary Materials. Tissues obtained from each individual were frozen in liquid nitrogen in the field and then transported to the campus of the University of California, Berkeley, where they were stored at −80°C until analysis. In addition, for a subset of localities, the Museum of Vertebrate Zoology at UC Berkeley provided tissue samples from individuals examined previously by Conroy and Neuwald (2008); detailed information regarding the sources of the tissue samples analyzed is provided in Appendix S1.

**Table 1:** Geographic location, number of individuals sampled (*n*), and origin relative to the mitochondrial contact zone for the 10 localities at which *Microtus californicus* were sampled during this study

	Locality	Longitude/Latitude	*n*	Assignment based on previous study^a^	Assignment based on present study
1	Castroville	36.7370/−121.7871	14	n/a	North clade
2	Rancho Marino	35.5379/−121.0883	10	North clade	North clade
3	Guadalupe	35.0280/−120.6160	12	n/a	North clade
4	AFB^b^	34.6881/−120.5879	14	n/a	North clade
5	Gaviota	34.4720/−120.2166	15	Contact zone	Contact zone
6	Refugio	34.4667/−120.0701	15	Contact zone	Contact zone
7	COPNR	34.4175/−119.8742	15	Contact zone	Contact zone
8	CSMR	34.3990/−119.5292	14	Contact zone	South clade
9	Ventura	34.1362/−119.1778	10	n/a	South clade
10	SJMR	33.6631/−117.8525	13	n/a	South clade

^a^Conroy and Neuwald (2008). ^b^Vandenberg Air Force Base.

### 2.2 Library preparation and sequencing

To examine introgression of the mitochondrial genome across the contact zone, we generated partial (264–789 bp) sequences for the cyt‐*b* locus. This portion of the cyt‐*b* locus was chosen in part because it matched sequences included in Conroy and Neuwald (2008). Cyt‐b sequences were obtained for a total of 132 *M. californicus*, all of which were also characterized using genome‐wide nuclear markers. Protocols for PCR‐amplification and sequencing of mitochondrial DNA followed those of Conroy and Neuwald (2008). Sequences were edited and aligned manually using Geneious Pro 5.1.7 (Kearse et al., 2012).

To provide a nuclear perspective on introgression across the contact zone, we used double digest restriction site‐associated DNA sequencing (ddRAD‐seq) as described by Peterson, Weber, Kay, Fisher, and Hoekstra (2012) to identify variable sites throughout the *M. californicus* genome. Details of the procedures used to develop genomic libraries are provided in the Supplementary Materials. Briefly, genomic DNA was extracted from samples of liver tissues obtained from 139 animals using a salt precipitation method (Miller, Dykes, & Polesky, 1988). DNA samples were digested using the restriction enzymes EcoR1 and SphI (NEB). Each digested sample was labeled via ligation to one of 24 unique adaptors. Between six and 24 of these uniquely labeled samples were pooled, resulting in eight libraries that were subject to size selection using the pippin prep procedure. We targeted fragment sizes from 376 to 476 bp (centered on ca. 426 bp), with an insertion size of 350 bp. Each library was then amplified using a distinct set of PCR‐indexing primers. Amplified libraries were pooled and were run on one lane of the Illumina Hiseq 4000 platform to generate 150 bp paired‐end sequence reads.

### 2.3 Data filtering and alignment

Raw sequence reads for all samples were de‐multiplexed and then cleaned by trimming adaptors and low quality reads following the protocols in Bi et al. (2012) and Singhal (2013). Cleaned sequences from each individual were mapped to a *M. californicus* reference genome (C. Conroy et al., unpublished data) using the program Novoalign (http://www.novocraft.com/products/novoalign/). Only reads that mapped uniquely to the reference genome were retained. Next, we used Picard (http://www.picard.sourceforge.net) to identify overlapping reads (read groups) and GATK (McKenna et al., 2010) to perform realignment with consensus RAD reads. We used SAMTools/bcftools to create a raw file in variant call format, after which we used SNPcleaner v.224 to remove low quality sites (e.g., low coverage, high percentage of missing data, putative paralogs) from our dataset (Bi et al., 2013). Additional information regarding these procedures is provided in the Supplementary Materials. Upon completion of these analyses, individuals exceeding a minimum threshold of 10× coverage were retained for further analyses.

### 2.4 Single nucleotide polymorphism and genotype calling

For analyses that required data to be input as discrete variants (e.g., ∂a∂i, cline analyses; see below), we identified single nucleotide polymorphism (SNP) markers contained in our genomic dataset. SNPs were identified and individual genotypes were assigned using ANGSD (Korneliussen, Albrechtsen, & Nielsen, 2014). Most of the analyses implemented in ANGSD were performed using likelihood estimates of per site allele frequencies, individual genotypes, or Bayesian posterior probabilities. For analyses of SNPs, we only considered sites for which likelihood ratio tests indicated that the SNP was variable (*p* ≤ 1e‐6). For analyses of genotypes, we only considered genotypic assignments with a posterior probability of ≥.95.

### 2.5 Determining phylogenetic relationships

To explore genetic differences between the northern and southern lineages of *M. californicus*, a phylogenetic tree based on the maximum‐likelihood optimality criterion was constructed using RAxML (Stamatakis, 2014). For additional insight, a second tree was generated using MrBayes 3.2.6 (Ronquist & Huelsenbeck, 2003). Both analyses were conducted based on the 132 cyt‐*b* sequences generated during this study plus sequences for *M. ochrogaster* and *M. mexicanus* that served as outgroup taxa (Table S1). Cyt‐*b* sequences were aligned using CLUSTAL W (Thompson, Higgins, & Gibson, 1994) as implemented in Geneious Pro 5.1.7 (Kearse et al., 2012). Details regarding tree construction are provided in the Supplementary Materials.

As an alternative approach to viewing cyt‐*b* sequence variation that allowed for reticulation, a statistical parsimony network was constructed using PopART (Leigh & Bryant, 2015). Additionally, a hierarchical analysis of molecular variance (AMOVA) was used to examine the distribution of cyt‐*b* nucleotide variation within and among lineages; this analysis was also conducted in PopART. Estimates of pairwise distances between cyt‐*b* sequences (uncorrected‐*p*) were generated using Mega 6.06 (Tamura, Stecher, Peterson, Filipski, & Kumar, 2013).

Finally, we examined relationships among individuals and lineages using the SNPs generated by our ddRAD‐seq dataset. We used ANGSD to identify SNP variants, after which we used SplitsTree4 (Huson & Bryant, 2006) to generate an unrooted neighbor‐joining tree depicting relationships among genotypes for individual samples.

### 2.6 Estimating divergence time

The divergence time between lineages based on cyt‐*b* sequences was estimated using BEAST v1.8.3 (Drummond, Suchard, Xie, & Rambaut, 2012). To provide a more robust perspective based on the evolutionary history of the genus *Microtus*, this analysis was conducted using an expanded dataset that included sequences used to generate our phylogenetic tree plus sequences from an additional eight clades of *Microtus* and *Myodes glareolus*; these additional sequences served as outgroup taxa (Table S1). No fossil or other reliable dates for divergence with North American *Microtus* have been reported (Chaline & Graf, 1988) and thus we used the established divergence time for European lineages of *M. agrestis* (Pauperio et al., 2012) to calibrate our analyses. The prior for the calibration node (the most recent common ancestor of the two *M. agrestis* lineages) was set to a lognormal distribution with a mean (±*SD*) value of 0.015 (±0.2) that incorporated the divergence date for the *M. agrestis* lineages. Additional details regarding the parameterization, assignment of prior values, and calibration of this analysis are provided in the Supplementary Materials.

### 2.7 Analyses of population‐level genetic variation

To explore potential evolutionary processes contributing to the maintenance of the putative contact zone between the northern and southern lineages of *M. californicus*, we used DnaSP v5.10.1 (Librado & Rozas, 2009) to generate estimates of multiple population‐level measures of mitochondrial haplotype diversity, including the number of haplotypes (*h*), nucleotide diversity (π), and Watterson's theta (θ*w*). We also used DnaSP to identify fixed differences and shared polymorphisms between mitochondrial lineages, as well as to conduct neutrality tests based on Tajima's *D* (Tajima, 1989), Fu' *F*s (Fu, 1997) and the *R*_2_ statistic (Ramos‐Onsins & Rozas, 2002). To assess mitochondrial haplotype differentiation among populations, we estimated *F*_ST_ across sampling localities; the significance of *F*_ST_ values was assessed using Arlequin v3.5 with 1,000 permutations (Excoffier & Lischer, 2010).

We also assessed population‐level diversity using admixture analyses (NgsAdmix, Skotte, Korneliussen, & Albrechtsen, 2013) of our ddRAD dataset; for these analyses, we used ANGSD to calculate the likelihood of assigning individuals to distinct genetic clusters. Analyses were performed for the entire dataset and, subsequently, for each of the genetic clusters identified within the complete dataset. The number of genetic clusters (*K*) was determined following Evanno, Regnaut, and Goudet (2005). We also used the maximum likelihood‐based option in ANGSD to generate a folded (unpolarized, no outgroup), population site frequency spectrum (SFS). We then used the estimated SFS to calculate mean genome‐wide values for per site π, θ_*w*_, and Tajima's *D* for each sampling locality and each genetic lineage. Additionally, the SFS was used to estimate *F*_ST_ between pairs of populations.

### 2.8 Characterization of the contact zone

To quantify the structure—the spatial distribution of genetic variation—across the putative contact zone, we conducted cline analyses using the *hzar* software package (Derryberry, Derryberry, Maley, & Brumfield, 2014) as implemented in R; these analyses were conducted using three different datasets. First, cline analysis was completed based on the frequencies of northern and southern cyt‐*b* haplotypes. Second, this analysis was conducted for individual SNPs identified as diagnostic. To identify diagnostic SNPs, we first used ANGSD to detect SNPs (*n* = 23,288 after exclusion of private alleles). We then calculated the average allele frequencies for each SNP among the three most northern populations and the two most southern populations sampled; these populations were targeted because earlier studies suggested that they are likely to represent pure parental lineages. SNP markers were considered to be diagnostic if the average allele frequency was >0.8 in one lineage and <0.2 in the other (adapted from Baldassarre, White, Karubian, & Webster, 2014); these semi‐arbitrary cutoffs were chosen to maximize the number of markers examined while retaining clear, diagnostic differences between parental lineages. Finally, we analyzed the average genomic cline based on the hybrid index (*Q*, the proportion of admixture from two parental genomes; Barton & Gale, 1993). The *Q*‐value for each individual was calculated based on data from diagnostic SNPs using INTROGRESS (Gompert & Buerkle, 2010).

### 2.9 Demographic inference

To explore potential impacts of the demographic histories of the northern and southern lineages on the dynamics of the putative contact zone, we used ∂a∂i (Gutenkunst, Hernandez, Williamson, & Bustamante, 2009; Robinson, Coffman, Hickerson, & Gutenkunst, 2014), which applies maximum‐likelihood methods to allele frequency spectra to evaluate potential historical demographic scenarios. We used the folded allele frequency spectrum (2D‐SFS) from the SNPs identified in this study as the input values for ∂a∂i. Because we were interested in assessing the demographic histories of the northern and southern lineages of *M. californicus*, we fit our data to two‐population models, in which each “population” was one of the lineages identified by our genome‐wide nuclear markers.

Because the results of our neutrality tests for cyt‐*b* (see Results) suggested possible historical changes in population size, our ∂a∂i analyses included models that allowed such changes. Initially, we evaluated our data against two null models: (1) no divergence of the ancestral population and (2) a change in size but no divergence of the ancestral population. Subsequently, we evaluated our data against six additional two‐population models representing different historical demographic scenarios. We chose these models for analysis because they captured the primary aspects of demographic history that we wanted to explore, namely level of gene flow, time of divergence, and historical changes in population size. Because no previous research regarding the demographic history of *M. californicus* was available*,* we explored all of the two‐population models built into ∂a∂i. Detailed descriptions of each model and the associated parameters are provided in Table 2. We ran each model five to ten times with random initial values for the parameters to be estimated, after which the best‐fit parameters for each model were determined by maximum‐likelihood estimation, with the best‐fit indicated when at least three fittings of that model converged on the same parameter set. To identify the best‐supported demographic model, we used the Akaike information criterion (AIC) (Akaike, 1998). Model selection was based on ΔAIC values (Burnham & Anderson, 2002). Once the best‐fit model had been selected, we used the uncertainty analysis in ∂a∂i to estimate the standard deviations for the associated demographic parameters. Details regarding the parameterization of each model and the uncertainty analyses are provided in the Supplementary Materials.

**Table 2:** Parameters used in evaluation of models of the demographic history of *Microtus californicus*. For each model considered, the input parameters are listed and a brief description of the model is provided. For parameter definitions, see Gutenkunst et al. (2009)

Model	Parameters	Description
Null models		
Standard neutral model (SNM)	None	Populations never diverge
Bottlegrowth (BG)	nuB, nuF, T	Instantaneous size change followed by exponential growth with no population split
Two‐population models		
Bottlegrowth_split	nuB, nuF, T, Ts	Instantaneous size change followed by exponential growth then split
Bottlegrowth_split_mig	nuB, nuF, m, T, Ts	Size change, exponential growth then split with migration
Split_mig	nu1, nu2, T, m	Split into 2 populations with specified size with migration
IM	s, nu1, nu2, T,m12, m21	Isolation‐with‐migration with exponential growth
IM_pre	nuPre, TPre, s, nu1, nu2, T, m12, m21	Isolation‐with‐migration with exponential growth, and a size change prior to split
Iso	s, nu1, nu2, T	Isolation then exponential growth

## 3 RESULTS

### 3.1 Data processing and variant calling

The raw data generated by the ddRAD‐seq procedure consisted of 80,791 Mb of sequence representing a total of 281,696,020 sequence reads (*n* = 139 individuals). After demultiplexing and removing adaptor sequences and low‐quality reads, 33,302 Mb of sequence remained in the cleaned dataset. Of those, 29,860 Mb (89.6%) mapped to the draft genome for *M. californicus*. Coverage per individual ranged from 4.6× to 159.9× (mean ± *SD* = 47.3 ± 32.1*X*). A total of 132 (95%) of the individuals sequenced were characterized by >10× coverage (mean ± *SD* = 48.68 ± 31.69*X*) and thus were retained in the dataset for subsequent analyses. From these animals, a total of 3,475,028 biallelic (biparentally inherited) sites were identified for use in analyses of genomic variability. From this dataset, a total of 56,343 SNPs were identified for potential use in analyses requiring discrete measures of genetic variation.

### 3.2 Phylogenetic relationships and lineage divergence

**Figure 2:**
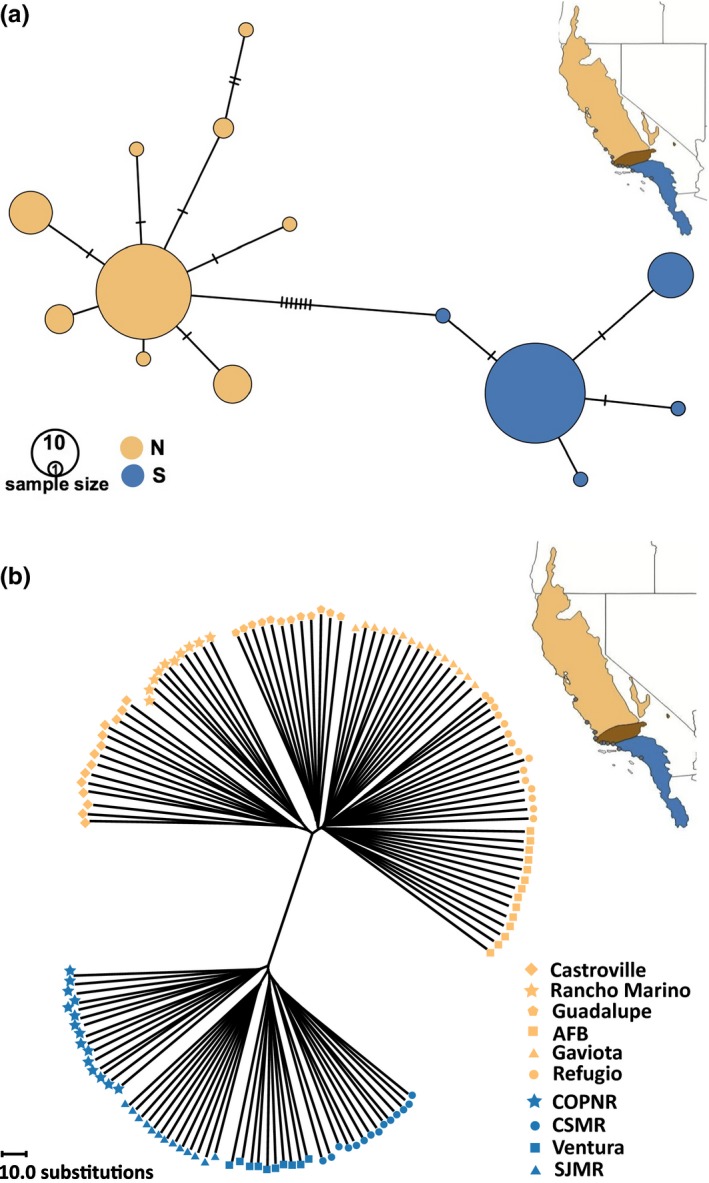
Genetic divergence of northern (orange) and southern (blue) lineages of *Microtus californicus*. All analyses are based on data from 10 to 15 individuals per locality for the 10 sampling localities shown in Figure 1. In (a), a statistical parsimony network for cyt‐*b* is shown. Each circle represents a distinct haplotype; the size of the circle denotes the relative frequency of that haplotype. Tick marks between circles indicate the number of mutations distinguishing those haplotypes. (b) An unrooted neighbor‐joining tree constructed from single nucleotide polymorphism is shown. Each branch represents a distinct genotype, with the population of origin indicated by the shape used to denote the genotype. Each symbol represents a single individual

Analyses of cyt‐*b* haplotypes were consistent with the previously reported (Conroy & Neuwald, 2008) occurrence of two well‐defined mitochondrial lineages among the populations sampled (Figures 2a and S1). Within each lineage, uncorrected‐*p* sequence divergence was ≤1%, while the difference between northern and southern mitochondrial lineages was ca. 4%; this difference in sequence divergence was statistically significant (Table 3, AMOVA, *p* = .006). There were seven fixed differences and no shared polymorphisms between the northern and southern mitochondrial lineages. Reciprocal monophyly of northern and southern lineages was well supported (Figures S1 and S2), with a mean estimated divergence time between lineages of 54.5 kya (95% HPD: 117.2–17.4 kya; Figure S2 and Table S2). The estimated substitution rate based on this divergence time was 0.5 per site per million years. A similar distinction between northern and southern genotypes was evident from analyses of SNP markers; an unrooted neighbor‐joining tree constructed from the SNP dataset revealed two distinct sets of genotypes that corresponded to the northern and southern mitochondrial lineages (Figure 2b).

**Table 3:** Hierarchical AMOVA for the mitochondrial cyt‐*b* locus. Relative haplotype variation within and among populations (sampling localities) as well as between the northern and southern lineages was assessed. Estimates of Φ are based on 1,000 permutations of the mitochondrial dataset

Variation	*df*	Sum of squares	σ^2^	% variation	Φ	*p* value
Among lineages	1	1,095.5	16.011	57.654	Φ_CT_ = 0.577	*p* = .006
Among populations within lineages	8	678.7	6.018	21.669	Φ_SC_ = 0.512	*p* < .001
Within populations	122	700.6	5.742	20.677	Φ_ST_ = 0.793	*p* < .001
Total	131	2,474.8	27.771			

### 3.3 Patterns of introgression across the contact zone

**Figure 3:**
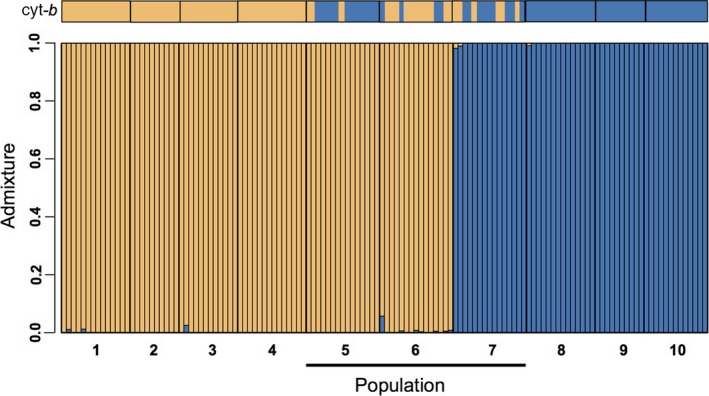
Haplotype distribution for the mitochondrial cyt‐*b locus* (upper panel) and admixture analysis based on probability of genotype assignment from all genomic sites (lower panel). The *x*‐axis depicts each individual sampled, with the population of origin (1–10; Table 1) indicated; the *y*‐axis denotes the probability of an individual (*N* = 132) being assigned to either genetic cluster. Analyses of nuclear markers resulted in two genetic clusters (*K* = 2). The black line highlights populations (5–7), which correspond to the putative contact zone. Although individuals in the contact zone displayed a mismatch between mitochondrial haplotype and genomic assignment, effectively no admixture was identified within the genomic dataset

The distribution of both mitochondrial haplotypes and nuclear genotypes across our sampling localities confirmed that the contact zone between the northern and southern lineages of *M. californicus* occurs in Santa Barbara County, CA (Figures 1 and 2). Patterns of introgression across the contact zone, however, differed markedly between our mitochondrial and nuclear datasets. With regard to mitochondrial markers, cyt‐*b* haplotypes from both northern and southern lineages were recovered in three populations (5‐Gaviota, 6‐Refugio, 7‐COPNR: Figure 1). In contrast, our admixture analysis based on genotypes assigned using all biallelic SNPs revealed two distinct, nonoverlapping genetic clusters (*K* = 2; Figure 3) with no clear indication of admixture. The break between these two genetic clusters occurred between populations 6 (Refugio) and 7 (COPNR), within the contact zone revealed by mitochondrial sequences. The nuclear data identified a few individuals that had a slight probability (≤1%) of being from the alternative lineage; given this low probability of admixture across the nuclear genome, it is not surprising that this result was not statistically supported by our SNP data. When both mitochondrial and nuclear DNA datasets were considered together, we detected a total of 22 individuals with mismatched mitochondrial haplotypes and nuclear genotypes in populations 5, 6, and 7 (Figures 1 and 3); mismatches did not extend to any of the other populations sampled. Thus, introgression of mtDNA was evident despite highly differentiated genomic backgrounds.

**Figure 4:**
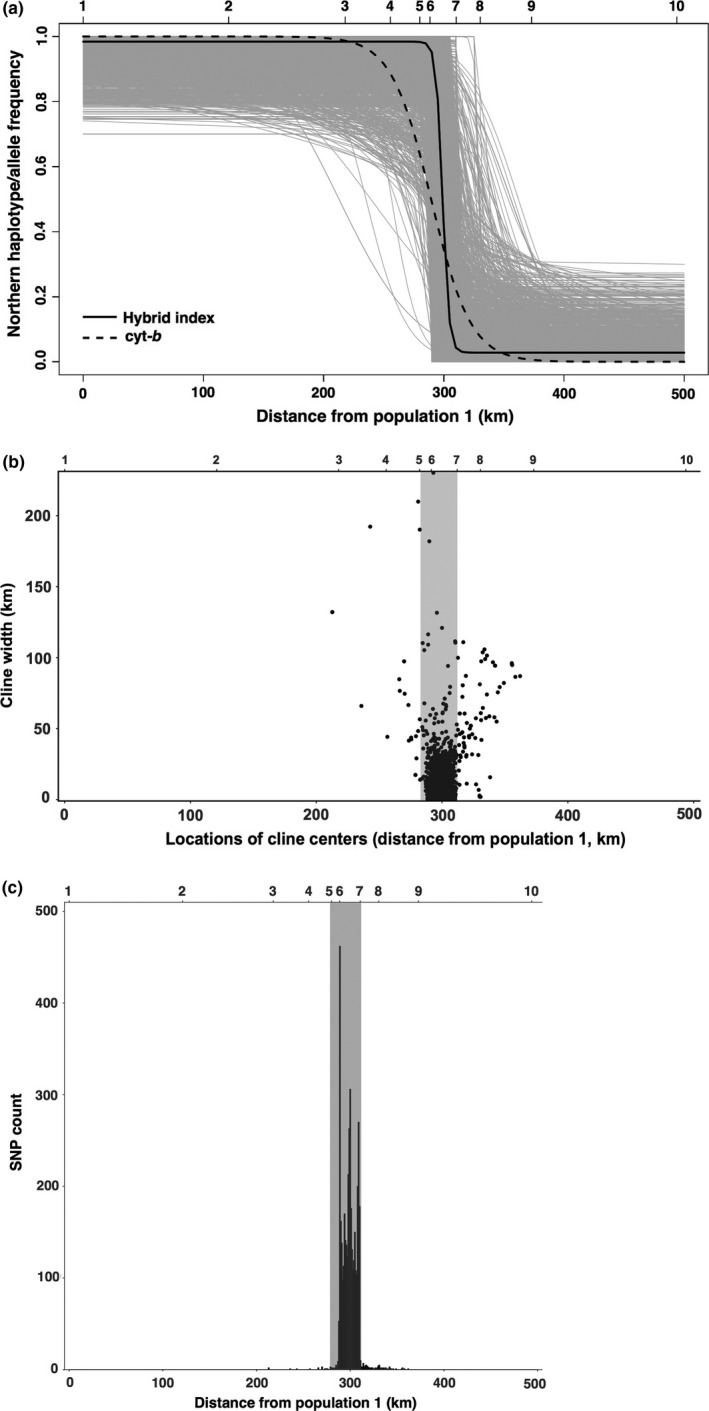
(a) Results of cline analyses for the mitochondrial cyt‐*b* locus (dashed line) and 4,050 diagnostic single nucleotide polymorphisms (SNPs) (gray lines), with the average genomic cline estimated from the hybrid index (*y*‐axis) represented by a solid black line. Numbers along the top of the figure denote the relative location of each population sampled (Table 1); the distance from population one for each subsequent population sampled is indicated along the *x*‐axis. (b) Distribution of estimates of cline center versus cline width for the 4,050 diagnostic SNP markers examined. The location of the contact zone (populations 5–7) is denoted with the gray rectangle. (c) Geographic distribution of cline centers for all diagnostic SNPs examined. Parameters indicated along the *x*‐axis (above and below figure) correspond to the two parameters indicated along the *y*‐axis in (a). Here, the *y*‐axis corresponds to the number of SNPs analyzed. The location of the contact zone (populations 5–7) is denoted with the gray rectangle

Consistent with these findings, the shape of the genetic cline between lineages differed markedly between mitochondrial and genomic markers. Cline analysis revealed that the center of the cyt‐*b* cline was located 289.42 km from population 1 (95% CI: 279.50–299.05 km), with a width of 65.99 km (95% CI: 44.37–104.5 km); this places the center of the mitochondrial cline between populations 6 and 7 (Figure 4). Individual clines were generated for each of the 4,050 diagnostic SNPs examined (Table S3); for the vast majority (97.8%) of these markers, cline centers also fell between populations 6 and 7 (Figure 4b,c). Cline analysis of hybrid index *Q*‐values revealed that the center of the average genomic cline was located 298.98 km from population 1 (95% CI: 298.43–299.05 km), with a width of 10.72 km (95% CI: 10.71–11.48 km); for 3,090 (76%) of SNP markers, the cline width was less than 7 km (Figure S5). Only 1.3% of SNPs displayed clines that were as wide or wider than the mitochondrial cline. Thus, while the geographic centers of clines for the two marker types were similar (distance from population1 = 289.42 km for mtDNA vs. 298.98 km for nDNA), the cline for the mitochondrial cyt‐*b* locus was considerably wider than that of the nuclear genome (mtDNA = 65.99 km; nDNA = 10.72 km).

For the 88 SNPs that did not have clines centered between populations 6 and 7, 31 (35.2%) of these markers had cline centers that fell within the geographic distribution of the northern lineage; the remaining 57 (64.8%) had cline centers that fell within the distribution of the southern lineage (Table S3). All animals (*n* = 45) in the three populations displaying evidence of mitochondrial introgression had *Q*‐values (proportion of the northern genotype) that were >0.9 or <0.1 (Figure S6), providing no genomic evidence of recurrent gene flow among sampling localities within or across the contact zone.

### 3.4 Genetic diversity, population differentiation, and departures from neutrality

Mitochondrial sequence data revealed that nucleotide diversity (π) and polymorphism (θ_*w*_) were greater in the northern lineage, although differences between lineages were not significant (Table 4). Values of π and θ_*w*_ based on the nuclear SFS were similar for both lineages (Table 4). Analyses of Tajima's *D* revealed no evidence of departures from neutrality at cyt‐*b* in either lineage; in contrast, Fu's *F*s was statistically different from neutral expectations in the northern lineage (Table 4). Estimates of Tajima's *D* based on nuclear data revealed no evidence of departure from neutral expectations in either lineage (Table 4).

**Table 4:** Comparisons of nucleotide diversity (π) and number of polymorphic sites (Watterson's θ) for the northern and southern lineages of *Microtus californicus*. Data from the mitochondrial cyt‐*b* locus and the nuclear SFS are shown; the sample size for each analysis (*n*) is indicated. The results of neutrality tests (Tajima's *D*, Fu's *F*s) conducted for each lineage are provided; significant departures from neutral expectations are indicated by asterisks

	North clade		South clade	
	Cyt‐*bn* = 70	Nuclear*n* = 80	Cyt‐*bn* = 62	Nuclear*n* = 52
π	0.003	0.005	0.002	0.004
θ	0.008	0.005	0.004	0.004
Tajima's *D*	−1.59	−0.53	−1.44	−0.32
Fu's *F*s	−5.02^a^	–	−2.43	–

SFS, site frequency spectrum.

a *p* < .01.

Within lineages, estimates of Tajima's *D* based on mitochondrial haplotypes revealed significant departures from neutrality in populations 1 (Castroville), 7 (COPNR), and 8 (CSMR) (Table S4). Estimates of *R*_2_ also revealed a significant departure from neutrality in population 7 (Table S4). The number of haplotypes (*h*) varied among the populations sampled but there were no conspicuous geographic patterns to this variation. Pairwise comparisons of populations revealed *F*_ST_ values based on mitochondrial haplotypes ranging from 0.00 to 1.00 (Table S6, bottom triangle). High *F*_ST_ values (e.g., *F*_ST_ > 0.5) were observed primarily between pairs of populations from different lineages. Exceptions occurred when (1) populations within the putative contact zone that were strongly biased toward one mitochondrial lineage were compared with populations from the other lineage or (2) populations within the same lineage containing large numbers of private mitochondrial haplotypes were compared. Consistent with these findings, AMOVA analyses revealed significant differences within and among populations as well as between lineages, with the greatest variation (57.7%) occurring between lineages (Table 3).

Analyses of genomic loci revealed the presence of two distinct genetic clusters (*K* = 2) within each lineage. In contrast to the apparent absence of gene flow across lineages, gene flow among populations within the same lineage was evident (Figure S3). Across all populations, values of π and θ_*w*_ ranged from 0.003 to 0.005; values of Tajima's *D* for individual populations revealed no evidence of departures from neutrality (Table S5). Pairwise *F*_ST_ values tended to be smaller for comparisons of populations within versus between lineages (Table S6, upper triangle). Thus, analyses of both mitochondrial and genomic markers indicated that populations from different lineages tended to be more genetically distinct than populations within the same lineage.

### 3.5 Inferences regarding demographic history

**Figure 5:**
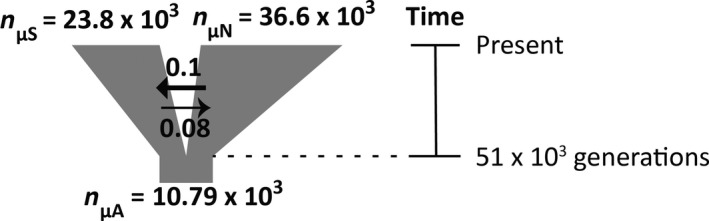
Summary of the demographic history of *Microtus californicus* as inferred from parameters estimated from the best‐fit model (isolation‐with‐migration with exponential population growth; Tables 2 and 4). The parameters *n*_μA_, *n*_μS_, and *n*_μN_ corresponded to population size of the ancestral population, the southern, and northern lineage, respectively. Arrows indicated the directions of gene flow

The results of our analyses of genetic differentiation within and among populations were used to inform the candidate models included in our simulations of demographic history. Although the majority of tests conducted failed to reveal evidence of significant departures from neutrality, estimates of Fu's *F*s, Tajima's *D* and *R*_2_ based on cyt‐*b* haplotypes suggested a possible historical change in population size (Tables 4 and S5). Thus, in addition to comparing empirical site frequency spectra (2D‐SFS) to models of no divergence between lineages, we also evaluated models that incorporated patterns of change in population size (no population size change, population expansion, reduction in population size due to a bottleneck). Comparisons of ΔAIC values (Table 5) indicated that although models IM and IM‐pre had similar maximum log‐likelihood values, model IM had the smallest AIC value (ΔAIC = 0), with the next best model, IM‐pre having a ΔAIC = 3.8. The difference in AIC values between these models suggests that model IM‐pre may over‐parameterize the data relative to model IM. The best‐fit demographic model—model IM—consisted of isolation‐with‐migration followed by exponential population growth; this model generated an estimated optimal θ = 750.68. Using a nucleotide substitution rate for house mice of 1.1 × 10^−8^ substitutions per site per generation (Drake, Charlesworth, Charlesworth, & Crow, 1998), the parameterization of this best‐fit model suggested that the ancestral population of *M. californicus* split between 51 kya (generation time = 1 year, Lidicker, 1976; Nelson, Dark, & Zucker, 1983) and 8.5 kya (generation time = 2 months, Cudworth & Koprowski, 2010; Greenwald, 1956), after which the two resulting daughter populations expanded in size to represent the current northern and southern lineages of this species, with limited migration between lineages (Table 6, Figures 5 and S7).

**Table 5:** Maximum log‐likelihood values, Akaike information criterion (AIC) scores, and ΔAIC values for the eight demographic models evaluated using our single nucleotide polymorphism dataset. Two null models (single population) and six‐two‐population demographic models were evaluated (Table 2). Abbreviations for models follow those used by the program ∂a∂i

Model^a^	Max. log‐likelihood	AIC score	ΔAIC
Null models			
Standard neutral model (SNM)	−156,101	312,202	>10
Bottlegrowth (BG)	−54,239	108,484	>10
Two‐population model			
Bottlegrowth_split	−3,079	6,166	>10
Bottlegrowth_split_mig	−1,707	3,412	>10
Split_mig	−1,811	3,630	>10
IM	−1,618.6	3,249.2	0
IM_pre	−1,618.5	3,253	3.8
Iso	−2,823	5,684	>10

a Model details described in Gutenkunst et al. (2009) and Robinson et al. (2014).

**Table 6:** Estimated demographic parameters for the two lineages of *Microtus californicus*. Data are from the best‐fit demographic model (isolation‐with‐migration and exponential population growth) identified by ∂a∂i, based on analyses of 2D‐SFS from 56,343 single nucleotide polymorphisms in our genomic dataset. For each parameter, the estimated value (±*SD*) is shown

Parameter^a^	Description^a^	Estimated value ± *SD*
s	Fraction of *n*_a_ that goes to N	0.64 ± 0.04
*n*_μN_	Final size of N	3.39 ± 0.7
*n*_μS_	Final size of S	2.21 ± 0.3
*T*	Time since divergence	2.35 ± 0.4
*m*_NS_	Migration (M) from S to N	0.08 ± 0.002
*m*_SN_	Migration (M) from N to S	0.1 ± 0.007

Units: *n*_μ_: Final effective population size; *T*: 2N generations; *m*: number of individuals (migration, M). SFS, site frequency spectrum; N, northern lineage; S, southern lineage.

a Gutenkunst et al. (2009).

## 4 DISCUSSION

Both mitochondrial and genome‐wide nuclear markers revealed a contact zone between the northern and southern lineages of *M. californicus* in Santa Barbara County, California. Although the location of this contact zone was consistent across marker types, the width of the zone differed substantially, with the cline for the mitochondrial cyt‐*b* locus being considerably wider than the average genomic cline. Consistent with this outcome, admixture analyses revealed little to no introgression of genomic loci and less than 20% of individuals exhibited mito‐nuclear mismatches in which a cyt‐*b* haplotype from one lineage was present against a nuclear background from the other lineage. The best‐fit demographic model identified by our analyses indicated that the northern and southern lineages are descended from a common ancestral lineage that diverged 8.5–51 kya. Collectively, these lines of evidence suggest that the current genetic structure of *M. californicus* reflects a major divergence event along the central coast of California, with little post‐divergence gene flow between the resulting genetically distinct lineages of voles.

### 4.1 Recent yet pronounced divergence in *Microtus*

Based on our analyses of mitochondrial sequences, the genetic differences between the two lineages of *M. californicus* reflect a relatively recent divergence event. The genus *Microtus* is thought to have undergone a rapid radiation within the last 1–2 million years (Fink, Fischer, Excoffier, & Heckel, 2010), with cyt‐*b* sequence differences among species typically ranging from 4% to 8% (Jaarola et al., 2004). Within the genus, sequence differences of 2%–6% have been linked to divergence events thought to have occurred within the last 20 kya (Barbosa et al., 2017; Bastos‐Silveira, Santos, Monarca, Mathias, & Heckel, 2012; Jaarola & Searle, 2004; Pauperio et al., 2012; Tougard, Brunet‐Lecomte, Fabre, & Montuire, 2008) and thus the 4% cyt‐*b* sequence differences and the estimated divergence time of ca. 54 kya for the northern and southern lineages of *M. californicus* are generally consistent with other studies of diversification in this genus, as is the estimated divergence time for these lineages based on SNPs (≤51 kya). Accordingly, evidence from both mitochondrial and genomic data indicates that the northern and southern lineages of *M. californicus* are the products of a recent divergence event. Interestingly, while the apparent timing of the cyt‐*b* divergence between lineages reported here is consistent with other analyses of divergence in *Microtus*, this level of sequence differentiation is greater than expected based on analyses of other mammal species (Suzuki et al., 2013; Tobe et al., 2010). Although it has been suggested that *Microtus* is characterized by a higher rate of molecular evolution than other mammalian taxa (Triant & DeWoody, 2006), the degree of divergence between the northern and southern lineages of *M. californicus* suggests that a focused, taxonomic re‐examination of these animals is warranted.

### 4.2 Discordance in patterns of introgression across the contact zone

Patterns of introgression across the contact zone differed markedly between mitochondrial and genomic markers. In particular, while admixture analyses revealed a lack of introgression at the genomic scale, introgression was evident for mitochondrial haplotypes; consistent with this, the cline width for cyt‐*b* was greater than that for most of the SNPs examined. Three lines of evidence suggest that this mito‐nuclear discordance reflects contact zone dynamics rather than incomplete sorting of recently diverged lineages. First, incomplete lineage sorting would be expected to result in a spatially random mixture of northern and southern mitochondrial haplotypes among local populations throughout the species' range, rather than restriction of admixture to populations located near the boundary of each lineage (McGuire et al., 2007; Toews & Brelsford, 2012). Second, incomplete lineage sorting should also impact the nuclear genome, resulting in a random geographic distribution of admixed nuclear genotypes. Finally, due to the shorter coalescent time of the mitochondrial genome, admixture of mitochondrial loci would not be expected to continue after sorting of the nuclear genome was complete. Thus, our data do not support incomplete lineage sorting as the primary explanation for the observed patterns of genetic diversity. Instead, we suggest that the introgression of mitochondrial haplotypes detected here reflects historical hybridization (Bastos‐Silveira et al., 2012; Good et al., 2008) between members of the northern and southern lineages, with minimal evidence of recurrent gene flow.

Several mechanisms may have contributed to the greater width of the mitochondrial cline (reviewed in Toews & Brelsford, 2012). For example, female‐biased dispersal may promote greater introgression of maternally inherited mitochondrial haplotypes (e.g., Ribeiro, Lloyd, Feldheim, & Bowie, 2012). Although dispersal in *M. californicus* is generally male‐biased (Cudworth & Koprowski, 2010), detailed consideration of dispersal dynamics suggests that at the leading edge of a population expansion, alleles moving from the established gene pool to the expanding gene pool are more likely to increase in frequency or to become fixed due to drift if gene flow *within* the expanding population is limited (Petit & Excoffier, 2009). As a result, mitochondrial haplotypes in the established gene pool will be more likely than nuclear alleles to increase in frequency because the greater movement of nuclear genes within the expanding pool reduces the potential for drift to act on these genes relative to mitochondrial loci (Petit & Excoffier, 2009). Assuming that a similar expansion dynamic characterizes either end of the contact zone for *M. californicus*, asymmetry in movement of genetic material may have contributed to the pattern of greater mitochondrial introgression observed in this study.

A second mechanism that may have contributed to the differential introgression of the mitochondrial and nuclear genomes is capture of the mitochondrial genome due to historical hybridization (McGuire et al., 2007), the probability of which is increased if specific haplotypes are associated with adaptation to local environments (e.g., Boratyński et al., 2011; Ribeiro, Lloyd, & Bowie, 2011; Tieleman, Williams, & Bloomer, 2003). In our study system, the contact zone between lineages of *M. californicus* is geographically concordant with a transition from colder, wetter environments in the north to warmer, dryer environments in the south (McGuire, 2010). As a result, it is possible that local adaptation has contributed to the preservation of specific mitochondrial haplotypes, regardless of nuclear background. Alternatively, introduction of foreign mitochondrial haplotypes may have resulted in the genetic rescue of local populations that had accumulated deleterious mutations due to Muller's ratchet (Sloan, Havird, & Sharbrough, 2017). Finally, under a model where mito‐nuclear incompatibility results in hybrid breakdown (Burton & Barreto, 2012; Burton, Pereira, & Barreto, 2013; Harrison & Burton, 2006), we would expect to see nuclear but not mitochondrial introgression. Instead, we see nearly no nuclear introgression and signatures of historical mitochondrial introgression, and thus mito‐nuclear incompatibility seems unlikely to explain our data. Future studies that explore the functional significance of mitochondrial haplotypes in *M. californicus* should prove useful in determining if the greater introgression observed for these markers reflects adaptive benefits associated with greater mitochondrial diversity or the retention of specific mitochondrial haplotypes.

### 4.3 Maintenance of a bimodal hybrid zone

The results of our cline analyses suggest that there is strong selection against hybridization between the northern and southern lineages of *M. californicus*. If introgression across a contact zone is a result of neutral diffusion, then the width of a genetic cline should be primarily a function of generation time since contact and dispersal rate (estimated as the root mean square dispersal distance) (Endler, 1977). The estimated dispersal rate for *M. californicus* is 75 m based on mark–recapture data from free‐living members of this species (Lidicker & Patton, 1987). To maintain the width of our mitochondrial cline (65.99 km) would require the passage of 123,274 generations (20,535–123,247 years, depending on generation time); to maintain the width of our mean genomic cline (10.72 km) would require the passage of 3,253 generations (542–3,253 years). The fossil record for *M. californicus* in the Santa Barbara County dates to 40,000–10,000 ybp (McGuire & Davis, 2013). If this temporal framework is accurate, it suggests that the width of our average genomic cline should be greater. Further, assuming that the cline for cyt‐*b* reflects neutral diffusion (Boratyński et al., 2011) with no selection against hybrids, then the width of the average genomic cline should be similar to the mitochondrial cline. This was not the case in our dataset, suggesting that neutral diffusion alone cannot explain the maintenance of mitochondrial and genomic clines between lineages of *M. californicus*.

Instead, the marked discrepancy between expected and observed cline widths revealed by our analyses suggests that maintenance of the contact zone in *M. californicus* reflects a dynamic balance between gene flow and selection against hybrids. More specifically, our data suggest that recurrent gene flow may be limited due to selection against hybrid individuals. The distribution of *Q* values for populations in the contact zone revealed a striking absence of hybrids, as expected for bimodal hybrid zones in which there is a strong barrier to gene flow (Jiggins & Mallet, 2000), a pattern that was retained when the stringency of our cline analyses was increased to consider only fixed diagnostic SNPs. Possible explanations for such a barrier include premating isolation or local adaptation to strongly differentiated environmental conditions (Gay et al., 2008; Jiggins & Mallet, 2000; Nosil et al., 2009). McGuire (2010) reported that variation in tooth morphology in *M. californicus* is correlated with climatic factors, raising the possibility that distinct lineages of this species have adapted to different local conditions. In laboratory crosses, Gill (1984) described aggressive interactions and a high rate of failure for between‐lineage crosses involving captive *M. californicus,* providing potential evidence for premating isolation. Alternatively, meiotic breakdown due to chromosomal asynapsis (Torgasheva & Borodin, 2016) may result in postzygotic isolation of lineages (Gill, 1984). Although none of these possibilities have been explored in detail, these observations suggest that multiple selective factors may have contributed to the absence of hybrids in the populations examined here. Future studies will investigate more explicitly the roles of these factors in maintaining this young contact zone.

### 4.4 Role of demographic history

Analyses of genomic markers revealed no overall evidence of departures from neutral expectations. In contrast, analyses of cyt‐*b* sequences indicated that patterns of haplotype variation in the northern lineage were not consistent with neutral expectations and that multiple populations located at the edge of the distribution of each lineage displayed significant departures from neutral expectations. This difference in outcomes between genomic and mitochondrial markers is perhaps not surprising given that the effective population size for the mitochondrial genome is half that of the nuclear genome, suggesting that mitochondrial markers are potentially more sensitive to evolutionary processes impacting genetic variation. Moreover, because most of the nuclear loci acquired from ddRAD‐seq are assumed to be neutral (Andrews, Good, Miller, Luikart, & Hohenlohe, 2016), analyses based on a large number of genomic markers may obscure signatures of selection at individual loci or signals of selection acting through polygenic adaptation (Pritchard, Pickrell, & Coop, 2010). As a result, it is perhaps not surprising that we detected greater evidence of departures from neutrality for our mitochondrial dataset.

The results of our demographic modeling confirmed the apparent role of historical population processes in shaping modern genetic diversity in *M. californicus*. In particular, demographic modeling suggested that since their initial split, both lineages grew exponentially, with growth being greater in the northern lineage. As a result, mitochondrial departures from neutrality detected in the northern lineage may simply reflect the greater magnitude of this historical demographic change. Overall, *F*_ST_ values calculated for genomic and mitochondrial data were larger across lineages, indicating that greater genetic differentiation between versus within lineages. Collectively, these findings suggest that following their initial split, the northern and southern lineages of *M. californicus* have experienced multiple demographic and evolutionary processes that have led to current patterns of genetic divergence. Future studies should explore the roles of selection and historical demographic changes in limiting current gene flow across the contact zone.

### 4.5 Phylogeographic diversification in *M. californicus*

Our data place the initial divergence between the northern and southern lineages of *M. californicus* (ca. 8.5–54 kya) within the Pleistocene, which encompasses the last glacial maxima in western North America (26.5–19 kya; Clark et al., 2009). Both the geologic and climatic histories of California suggest that changes in land features and habitats during the Pleistocene were unlikely to have produced physical barriers that would have halted gene flow between these lineages of *M. californicus* (McGuire & Davis, 2013; Wigand, 2007). Of the primary phylogeographic breaks documented in California (Davis, Koo, Conroy, Patton, & Moritz, 2008; Jacobs, Haney, & Louie, 2004; Rissler, Hijmans, Graham, Moritz, & Wake, 2006; Schierenbeck, 2014), the Transverse Ranges are located in closest proximity to the contact zone for *M. californicus*. The Transverse Ranges, which sit at the junction of the Sierra Nevada Mountains, the Coast Ranges, and the Mojave Desert, are one of the few west‐to‐east oriented mountain ranges in California (Davis et al., 2008; Schierenbeck, 2014). This region, which includes parts of Santa Barbara County, encompasses diverse habitats and includes phylogenetic boundaries for several lineages of vertebrates (Burns & Barhoum, 2006; Davis et al., 2008; Matocq, 2002; Shaffer, Pauly, Oliver, & Trenham, 2004; Spinks & Shaffer, 2005). While these features suggest that the Transverse Ranges are an important barrier to gene flow—particularly along the more environmentally diverse inland side of this mountain range (Davis et al., 2008)—the rise and rotation of the Transverse Ranges occurred in the mid‐Pliocene (Nicholson, Sorlien, Atwater, Crowell, & Luyendyk, 1994), well before the apparent divergence of the northern and southern lineages of *M. californicus*. Currently, this species occurs at elevations ranging from sea level to ~2,700 m (Kellogg, 1918), a distribution that encompasses the highest portions of the Transverse Ranges. As a result, despite the overall importance of the Transverse Ranges to the biogeography of California (Calsbeek et al., 2003), it seems unlikely that these mountains have contributed to the maintenance of the distinct lineages of *M. californicus* studied here (see also McGuire & Davis, 2013).

With regard to potential environmental barriers, the proposed initial separation of the northern and southern lineages of *M. californicus* within the past 100,000 years coincides with a period during which central California was characterized by cooler, wetter conditions that would have facilitated the occurrence of the grassland and meadow habitats (Wigand, 2007) preferred by this species (Batzli & Pitelka, 1971; Cockburn & Lidicker, 1983; Cudworth & Koprowski, 2010). Low sea levels along the central coast of California during this period should also have resulted in expansion of grassland habitats (Rick, Erlandson, Jew, & Reeder‐Myers, 2013), presumably promoting migration and introgression rather than contributing to the formation of distinct genetic lineages of voles. Further research is needed to elucidate the environmental factors that contributed to the diversification of *M. californicus* in central California. In particular, analyses that incorporate detailed studies of habitat use and physiological tolerances will likely generate insights into the combination of conditions that favored this divergence event.

## 5 CONCLUSIONS

We investigated the occurrence of a putative contact zone between genetically distinct northern and southern lineages of *M. californicus* in the vicinity of Santa Barbara County, California (Conroy & Neuwald, 2008). Both mitochondrial and genome‐wide nuclear markers confirmed the presence of this contact zone, which appeared to reflect a relatively recent divergence event within *M. californicus*. Both the width of the contact zone and evidence for introgression among lineages differed between the molecular markers examined. In particular, while the mitochondrial dataset revealed some evidence of introgression across the contact zone, nuclear markers provided no evidence of recurrent gene flow between lineages, suggesting that the introgression of mitochondrial haplotypes reflects one or more historical hybridization events. Demographic modeling based on our genomic data also supported this scenario. Reasons for the initial separation of these lineages remain unclear, as neither conspicuous geographic nor environmental barriers to gene flow appear to have existed in the vicinity of the contact zone during the past 100,000 years, when this separation is thought to have occurred. Future studies, including analyses of habitat use, sensory ecology, reproductive isolation, and historical phylogeography, are required to elucidate fully the factors contributing to the divergence of these lineages and, more generally, to enhance understanding of how neutral and selective processes interact to maintain boundaries among genetic lineages of vertebrates.

## DATA ACCESSIBILITY

Raw read data are available at the National Center for Biotechnology Information Sequence Read Archive (Bio project no. PRJNA432693). Mitochondrial cyt‐*b* sequences are available on GenBank (accession no. MG916839‐MG916898).

## CONFLICT OF INTEREST

None declared.

## AUTHOR CONTRIBUTIONS

D. Lin, E. Lacey, and R. Bowie designed research. D. Lin and C. Conroy performed research. K. Bi, C. Conroy, and J. Schraiber contributed to analytical tools. D. Lin, K. Bi, and J. Schraiber analyzed the data. D. Lin, K. Bi, C. Conroy, E. Lacey, J. Schraiber, and R. Bowie wrote the manuscript.
