# Supplementary material for: Mito‐nuclear discordance across a recent contact zone for California voles
Source: Ecol Evol. 2018 May 24;8(12):6226–41. doi: 10.1002/ece3.4129 (PMC6024151; doi:10.1002/ece3.4129)
Supplement: Supplementary file 1 [file ECE3-8-6226-s001.pdf]

**Mito-nuclear discordance across a recent contact zone for California voles**

**Dana Lin, Ke Bi, Christopher Conroy, Eileen A. Lacey,**
**Joshua G. Schraiber and Rauri C. K. Bowie**

## Supplementary Document

## Materials and Methods

*Field collection of Samples*

All animals were live-trapped using Sherman traps baited with oats and peanut butter; captured individuals were euthanized via overdose of Isoflurane. Voucher specimens (skulls, skeletons, study skins) prepared from these animals were deposited in the Museum of Vertebrate Zoology (MVZ) at UC Berkeley (Appendix I). All procedures involving live animals were approved by the Berkeley Animal Care and Use Committee and conformed to the guidelines of the American Society of Mammalogists for the use of wild mammals in research (Sikes *et al.* 2016).

*Library preparation and sequencing*

Libraries were prepared in the Evolutionary Genetics Laboratory at the Museum of Vertebrate Zoology. We followed the ddRAD protocol of Peterson *et al.* (2012). After digestion, 24 unique P1 adaptors (forward: CACTCTTTCCCTACACGACGCTCTTCCGATCT-GCATxxxxx; reverse: /5Phos/xxxxxAGATCG-GAAGAGCGTCGTGTAGGGAAAGAGTGT; "xxxxx" represents a unique nucleotide sequence used as an internal barcode) that bind to SphI restriction sites were ligated to each sample. P2 adaptors (forward: TGACTGGAGTTCAGACGTGTGCTCTTCCGATCT; reverse: /5Phos/AATTAGATCGGAAGA-GCGAGAACAA) that bind to EcoR1 restriction sites were ligated to all individuals. Six to 24 uniquely-labeled samples were pooled as one library. Pooled samples were then subjected to the pippin prep procedure for size selection. After multiplexing, 8 libraries were submitted for Illumina sequencing at the Vincent J. Coates Genomics Sequencing Laboratory at UC Berkeley (supported by NIH S10 OD018174 Instrumentation Grant).

*Data filtering and alignment*

Raw fastq reads for all samples were first de-multiplexed based on the internal barcodes applied during library construction, with a maximum tolerance of one mismatched basepair per barcode. De-multiplexed reads were removed if the expected restriction enzyme cutting sites were not found at the beginning of the 5'-end of the read. The resultant sequences were then filtered using Skewer (Jiang *et al.* 2014) and Trimmomatic (Bolger *et al.* 2014) to remove adaptor sequences and low quality reads. Next, to remove potential bacterial contamination, we aligned reads to the *Escherichia coli* genome with Bowtie2 (Langmead & Salzberg 2012). After removing potential contaminant sequences, overlapping paired reads were merged with Flash (Magoc & Salzberg 2011). Clean paired and merged sequences from each individual were then mapped to a reference genome for *Microtus californicus* (Conroy et al., unpubl. data) with Novoalign

(http://www.novocraft.com/products/novoalign/). Only reads that mapped uniquely to the reference genome were retained.

We then used Picard (http://www.picard.sourceforge.net) and GATK (McKenna *et al.* 2010) to perform realignment on alignment files in BAM format, as generated by SAMtools (Li *et al.* 2009). We then used SAMTools/bcftools (Li *et al.* 2009) to generate a raw variant call format (VCF) file that contained all potential variable and invariable sites. Using the VCF file, we calculated the mean coverage for each individual; only individuals with $\geq$ 10 X coverage were retained in the dataset. To remove low quality sites (e.g. low coverage, high percentage of missing data, putative paralogs) from our dataset, data in VCF format were filtered using SNPcleaner (v.224, https://github.com/fgvieira/ngsClean) following the protocol of Bi et al. (2013). The specific flags used in SNPcleaner were –k 97 ($\geq$ 70% of our sample size), -u 3 (minimum read depth coverage of each individual at a site), -d 292 (minimum total read depth at a site), -h 0 (minimum p-value for a Hardy-Weinberg Exact test), and –H 0.0001 (minimum p-value to test for heterozygosity excess). This bioinformatics pipeline recovered a total of 3,475,028 sites, including variant and non-variant sites.

*Calling SNPs and genotypes and related analyses*

The procedures employed to call SNPs and assign genotypes are described in the primary text. For the NgsAdmix analysis, all of the 3,475,028 sites identified were used to calculate the likelihood of assigning an individual to a given genetic cluster. Values of –K (number of ancestral populations) ranged from one to ten; we specified -minMaf (the minimum minor allele frequency) to be 0.0038 and –minInd (minimum number of informative individuals) to be 92. Using these settings, we completed 20 runs of NgsAdmix analysis, after which we employed the Evanno et al. (2005) method to determine the optimal value of K. For NgsAdmix analysis *within* each clade, the –minMaf and –minInd were adjusted according to the number of voles sampled within each clade.

From the full data set of > 3 million genomic sites, we used ANGSD to identify 56,343 SNPs. These SNPs were used to generate an unrooted neighbor-joining tree depicting relationships among individual nuclear genotypes. The same markers were used in demographic models constructed using the program, ∂a∂i. These markers were analyzed using the POPFilter module in PopGenTools (https://github.com/CGRL-QB3-UCBerkeley/PopGenTools) to exclude private alleles, with flags set as: –i 0.2 (missing data allowed per sub-population) and –s 0.2 (total missing data allowed). We then used the realSFS sub-program in ANGSD to select consensus sites that existed across all populations, after which customized scripts were employed to calculate allele frequencies for each SNP and to filter out non-diagnostic SNPs using the cut-off rationale outlined in the Materials and Methods section of the primary text. After completion of these procedures, the resulting dataset contained 4,050 diagnostic SNPs that were used for analyses of cline structure and for calculations of hybrid index (Q) values.

*Phylogenetic analysis*

The maximum-likelihood tree depicting relationships among *cyt-b* haplotypes was generated with RAxML and was based on 1,000 rapid bootstrap replicates conducted through the CIPRES supercomputing facility (Miller *et al.* 2010). Additionally, we used jModelTest2 (Darriba *et al.* 2012; Guindon & Gascuel 2003) to identify the best-fit model of nucleotide substitution (F81+I+G) for our dataset. This information was used to inform the settings employed in MrBayes 3.2.6; two runs of MrBayes were completed, with each run consisting of 4 chains of 3 million generations, with sampling conducted every 100 generations.

For the BEAST analysis used to date the putative divergence event between lineages of *M. californius*, a data matrix consisting of one *cyt-b* sequence from each locality sampled (all 10 sequences were 789 bp) as well as sequences from representatives from other clades of *Microtus* and the *Myodes* outgroup was subjected to two independent MCMC runs, with chain lengths of 160 million generations each. Burn-in was set at 10% and output parameters were recorded every 16,000 generations. We used the HKY substitution model with estimated base frequencies and an uncorrelated, relaxed molecular clock. The tree prior was set to employ the Yule process. We forced the analysis to fit four monophyletic groups (the genus *Microtus*, all North American *Microtus*, the two *M. agretis* lineages and the two *M. californicus* lineages) based on previous studies that strongly support the monophyly of each of these lineages (Conroy & Cook 2000; Fink *et al.* 2010; Jaarola *et al.* 2004). Calibrations were placed at the node for the most recent common ancestor of the two *M. agrestis* phylogenetic lineages in Europe (Pauperio *et al.* 2012). The node prior was set to a lognormal distribution with a mean value of 0.015 and a Stdev of 0.2. The ucld mean prior was set to a gamma distribution with shape = 0.01 and scale = 1,000. The prior for Yule.birthRate was also set to a gamma distribution with the same shape and scale values. Default settings were used as the priors for the rest of the model parameters. Output trees from these runs were combined (10% burn-in) to generate a maximum clade credibility tree using the LogCombiner and Tree Annotator modules in BEAST v.1.8.3. *Cyt-b* sequences did not provide informative markers for resolving deep nodes in *Microtus* (Fink *et al.* 2010) and thus we did not attempt to determine the divergence dates for deep nodes in the trees generated by our analyses. Instead, we used these analyses to explore the likely dates for more recent divergence events, which are likely to be similar for *M. agrestis* and the lineages of *M. californicus* that are the focus of this study.

*Demographic model fitting using ∂a∂i*

Demographic analyses were conducted using the program *∂a∂i*; the parameters used in these analyses are given in Table 2. The input SFS was projected to a sample size of N = 50 for each lineage (50,50). The maximum number of iterations for each model was 20. In general, the upper bounds for population size were set to between 10 and 25. Size change upper bound was set to 5 except for the model in which this value is a fraction of original population size. Time since a lineage split or population size change had upper bounds between 10 and 15. Upper bounds for migration were set to between 2 and 5. The lower bounds for the parameters mentioned above were set between $1x10^{-2}$ - $1x10^{-100}$. For the uncertainty analysis, we bootstrapped the original SNP dataset to generate 100 sets that were equal in the number of SNPs in the original dataset. We then used these 100 simulated datasets to run uncertainty analyses using the Godambe method (Coffman *et al.* 2016) as implemented in *∂a∂i*.

**Table S1**. Outgroup taxa used in the analyses of divergence time estimation in *M. californicus*. *Cyt-b* sequences for these taxa were obtained from GenBank; the accession number for each sequence is indicated.

| Outgroup species | GenBank number |
|---|---|
| *Myodes glareolus* | AY309421.1 |
| *Microtus ochrogaster* | NC_027945.1 |
| *Microtus mexicanus* | AF163897.1 |
| *Microtus agrestis* FRANCE | JX284283.1 |
| *Microtus agrestis* SPAIN | JX284279.1 |
| *Microtus pennsylvanicus* | AF119279.1 |
| *Microtus montanus* | KF948532.1 |
| *Microtus longicaudus* | KF964335.1 |
| *Microtus pinetorum* | AF163904.1 |

**Results**

**Table S2**. Mean estimated divergence dates, 95% highest posterior density interval (HPD) and the posterior probability between selected lineages of *Microtus* and between *Microtus* and the *Myodes* outgroup. Estimates are from the maximum clade credibility tree for *Microtus* generated using BEAST. Node numbers correspond to nodes depicted in Figure S2.

| **Node** | **Mean (Mya)** | **95%HPD** | **Pos. prob.** |
|---|---|---|---|
| (1) *Microtus/Myodes* | 0.314 | (0.111, 0.654) | 1 |
| (2) *Microtus* | 0.286 | (0.104, 0.586) | 1 |
| (3) *M. agrestis* N/S clades | 0.0141 | (0.009, 0.020) | 1 |
| (4) *M. mexicanus/ M californicus* | 0.180 | (0.021, 0.373) | 0.979 |
| (5) *M. californicus* N/S clades | 0.0545 | (0.017, 0.117) | 1 |

**Table S3.** The parameters estimated by the cline analysis for each of the 4,050 diagnostic SNPs. Results are presented in the associated csv file for ease of viewing.

**Table S4**. Number of haplotypes (h), nucleotide diversity (π), and Watterson's θ (from number of segregating sites) estimated per-site from *cyt-b* sequences obtained from each sampling locality. Values of Tajima's D and Ramos-Onsins & Rozas's $R_2$ statistics are also shown. Asterisks denote significant departures from neutral expectations.

| Population | **1** | **2** | **3** | **4** | **5** | **6** | **7** | **8** | **9** | **10** |
|---|---|---|---|---|---|---|---|---|---|---|
| h | 3 | 3 | 1 | 8 | 3 | 5 | 2 | 4 | 1 | 5 |
| π | 0.001 | 0.001 | 0 | 0.003 | 0.01 | 0.02 | 0.019 | 0.006 | 0 | 0.002 |
| θ | 0.002 | 0.002 | 0 | 0.004 | 0.01 | 0.01 | 0.011 | 0.01 | 0 | 0.002 |
| Tajima's D | -1.95* | -0.36 | N/A | -0.8 | 0.27 | 1.25 | 2.52** | -1.93* | N/A | 0.08 |
| $R_2$ | 0.17 | 0.17 | N/A | 0.10 | 0.16 | 0.20 | 0.26** | 0.17 | N/A | 0.20 |

* $p<0.05$, ** $p<0.01$, *** $p<0.001$

**Table S5**. Average nucleotide diversity (π), Watterson's θ and Tajima's D. Data are from per-site estimates calculated across all genomic markers (averaged from all scaffolds) for each sampling locality.

| Population | **1** | **2** | **3** | **4** | **5** | **6** | **7** | **8** | **9** | **10** |
|---|---|---|---|---|---|---|---|---|---|---|
| π | 0.004 | 0.004 | 0.004 | 0.004 | 0.004 | 0.004 | 0.003 | 0.003 | 0.003 | 0.003 |
| θ | 0.005 | 0.004 | 0.004 | 0.004 | 0.004 | 0.004 | 0.003 | 0.003 | 0.003 | 0.003 |
| Tajima's D | -0.21 | 0.58 | 0.29 | 0.19 | 0.48 | 0.26 | 0.31 | 0.39 | 0.48 | 0.22 |

**Table S6**. Pairwise estimates of $F_{ST}$ for the localities sampled. The upper triangle depicts $F_{ST}$ values estimated from the nuclear SFS; the bottom triangle depicts $F_{ST}$ values estimated from *cyt-b*. Numbers 1-10 represent each sampling locality, as identified in Table 1. Populations 5, 6, and 7 contained both northern and southern *cyt-b* haplotypes. Grey cells denote comparisons between localities representing different clusters of nuclear genotypes, as revealed by admixture analyses. Permutation tests to assess the statistical significance of $F_{ST}$ was conducted only for the *cyt-b* dataset; bold Fst values were significantly different from 0.

| | **1** | **2** | **3** | **4** | **5** | **6** | **7** | **8** | **9** | **10** |
|---|---|---|---|---|---|---|---|---|---|---|
| **1** | | 0.14 | 0.14 | 0.14 | 0.16 | 0.16 | 0.55 | 0.56 | 0.56 | 0.58 |
| **2** | 0 | | 0.15 | 0.16 | 0.18 | 0.18 | 0.58 | 0.60 | 0.60 | 0.61 |
| **3** | 0.01 | 0.2 | | 0.07 | 0.10 | 0.10 | 0.57 | 0.58 | 0.58 | 0.60 |
| **4** | 0.04 | 0.11 | 0.05 | | 0.06 | 0.06 | 0.56 | 0.58 | 0.58 | 0.60 |
| **5** | **0.76** | **0.71** | **0.77** | **0.76** | | 0.05 | 0.57 | 0.58 | 0.59 | 0.61 |
| **6** | **0.19** | 0.12 | **0.19** | 0.18 | **0.40** | | 0.56 | 0.57 | 0.58 | 0.60 |
| **7** | **0.54** | **0.46** | **0.55** | **0.54** | 0.04 | **0.16** | | 0.12 | 0.15 | 0.16 |
| **8** | **0.93** | **0.91** | **0.94** | **0.93** | 0.09 | **0.64** | **0.29** | | 0.16 | 0.17 |
| **9** | **0.98** | **0.98** | **1** | **0.98** | **0.53** | **0.69** | **0.48** | **0.78** | | 0.16 |
| **10** | **0.98** | **0.98** | **1** | **0.98** | 0.13 | **0.69** | **0.34** | 0.01 | **1** | |

**Table S7.** Centers and widths of the average genomic clines estimated with relaxed and strict cut-off for defining diagnostic SNPs, and those of the *cyt-b* cline.

| | Center | 95% CI of center | Width | 95% CI of width |
|---|---|---|---|---|
| **HI cline (cutoff 0.8/0.2)** | **298.98** | **298.43-299.05** | **10.72** | **10.71-11.8** |
| **HI cline (cutoff 1/0)** | **298.7** | **298.66-298.7** | **5.07** | **5.07-5.08** |
| ***cyt-b* cline** | **289.42** | **279.50-299.05** | **65.99** | **44.37-104.5** |

**Figure S1.** Maximum-likelihood tree for mitochondrial *cyt-b* sequences from 132 *M. californicus* sampled during this study; data from 2 outgroup taxa (*M. mexicanus* and *M. ochrogaster*) are also included. Analyses were conducted using RAxML; relationships among main branches were identical for a Bayesian tree reconstructed using MrBayes. Numbers above branches support values from ML bootstrap/Bayesian posterior probabilities. Sequences associated with the southern versus northern clades are indicated.

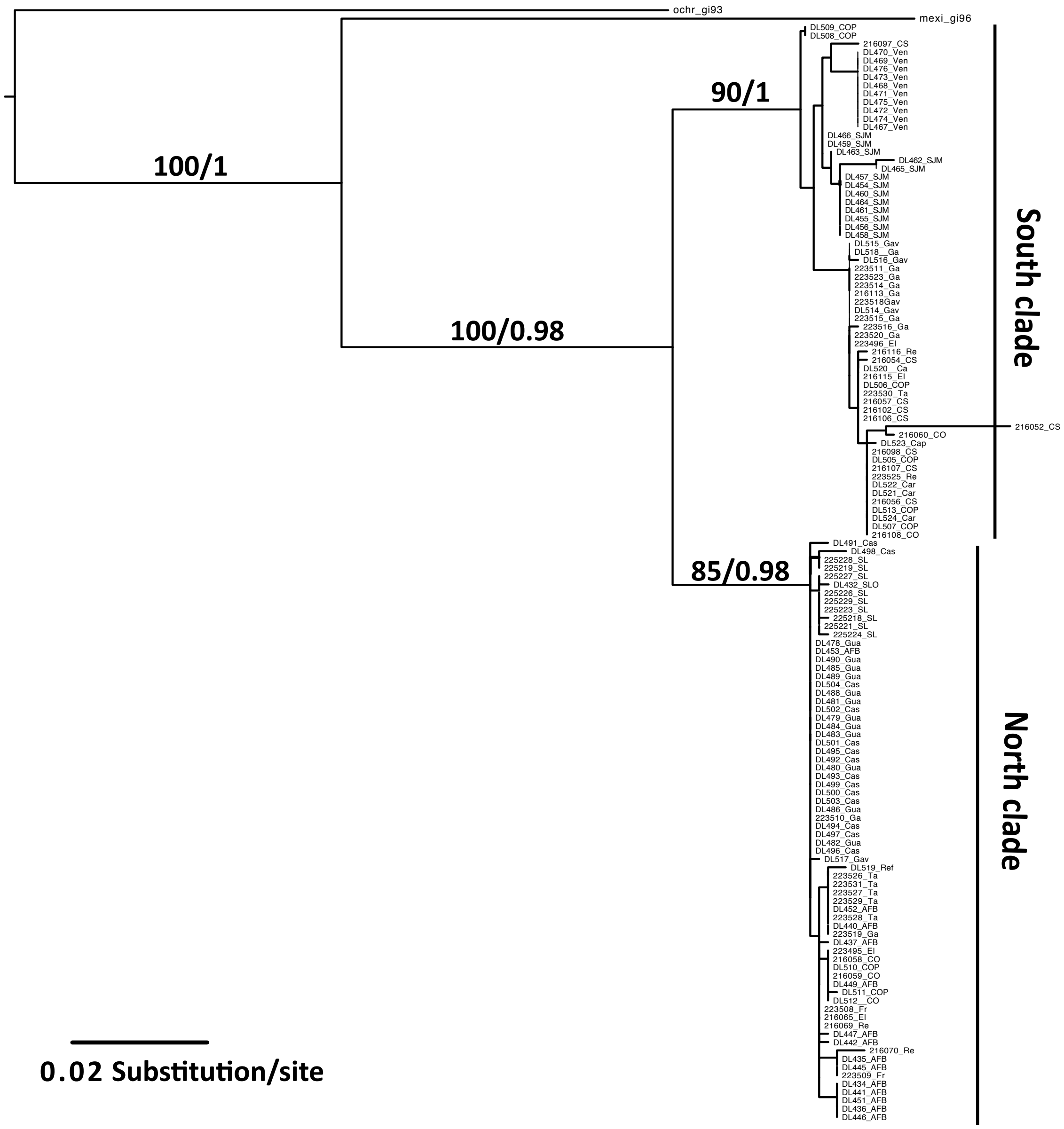

**Figure S2.** Maximum clade credibility tree depicting the estimated divergence time between the northern and southern lineages of *M. californicus*. Analyses were conducted for *cyt-b* sequences using BEAST. Nodes labeled with dots have Bayesian posterior probabilities greater than 0.95. The node numbers correspond to those indicated in Table S3, which provides the estimated divergence date for each node of interest.

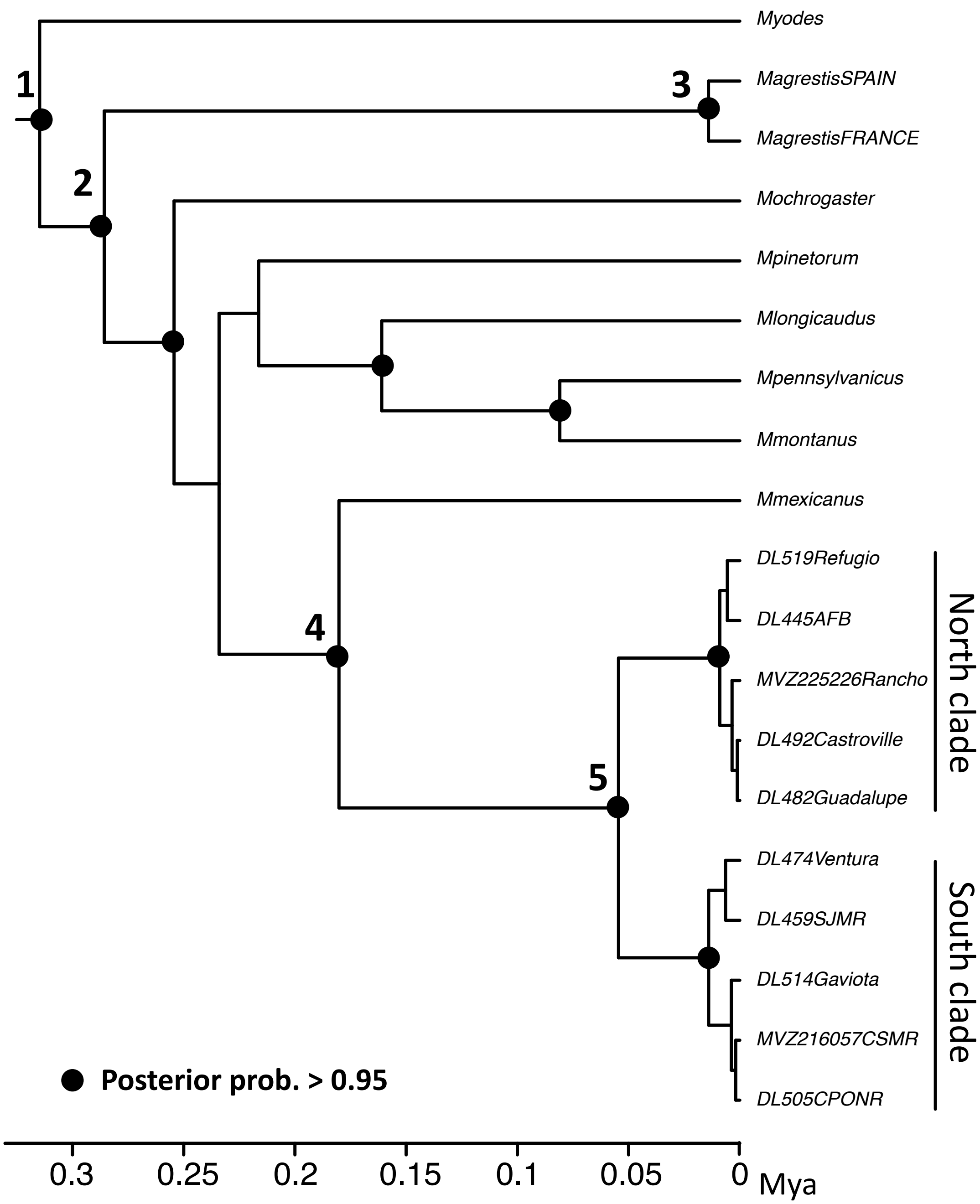

**Figure S3.** Results of admixture analyses for sampling localities within (a) the northern lineage and (b) the southern lineage of *M. californicus*. Analyses for each lineage were conducted separately to emphasize fine-scale genetic structure among sampling localities. For both lineages, the analysis indicated that K=2. Population numbers along the bottom of each panel correspond to the sampling localities listed in Table 1.

(a)

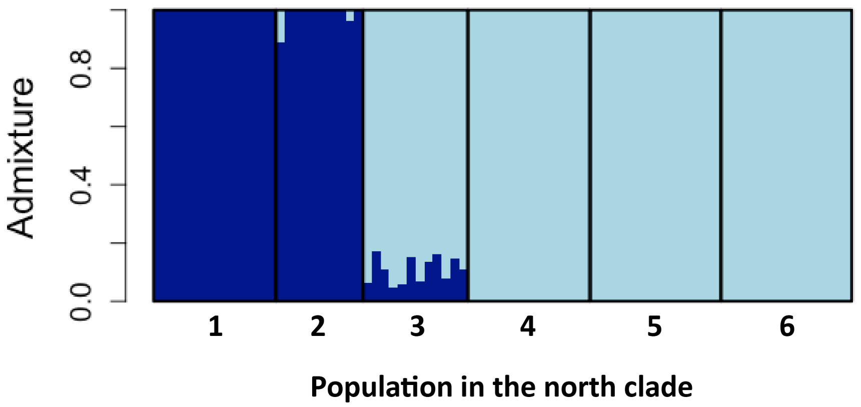


(b)

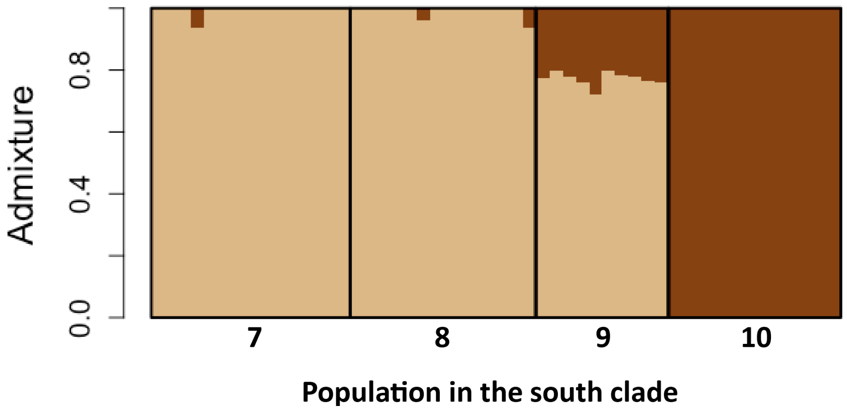

**Figure S4.** (a) Geographic distribution of the cline center for each diagnostic SNP. Data from a total of 4,050 SNPs are shown. Each point represents a single SNP marker; the 95% CI for each estimated cline center is indicated with the vertical bars. The y-axis to the left of the figure depicts absolute distance from sampling locality 1; the y-axis on the right indicates the relative location of each sampling locality. Sampling locality numbers correspond to those in Table 1.

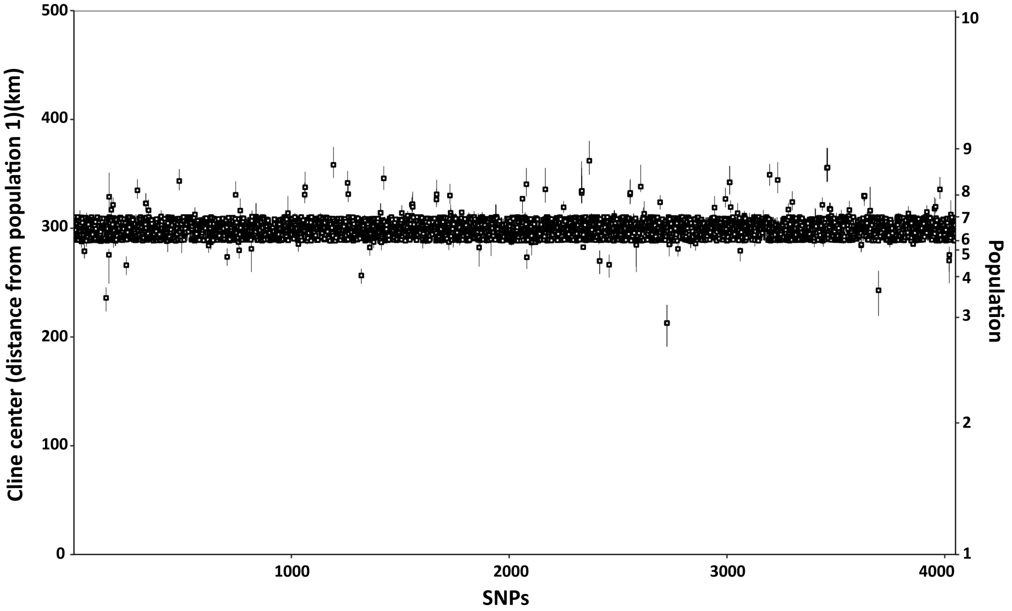


**Figure S5.** Distribution of cline widths for all diagnostic SNPs examined.

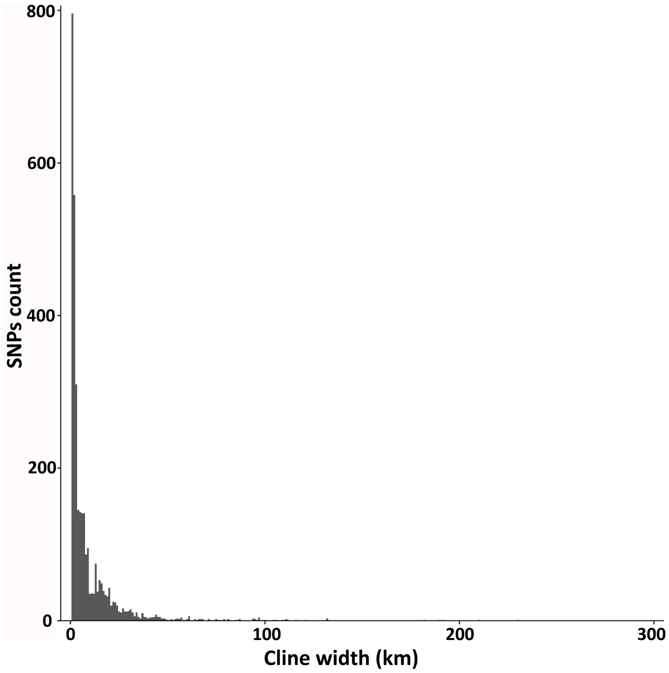

**Figure S6.** Distribution of hybrid index (Q) scores for individual diagnostic SNPs analyzed for three sampling localities (5-Gaviota, 6-Refugio and 7-COPNR) located within the contact zone for *M. californicus.*

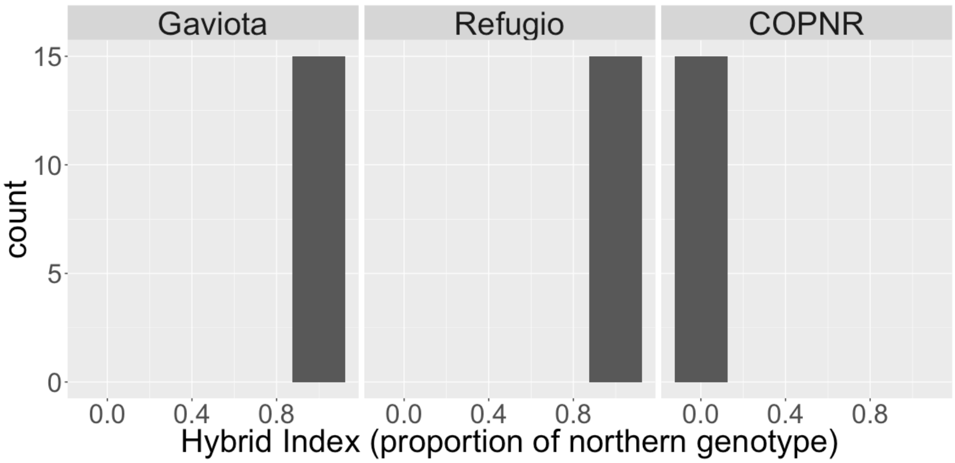


**Figure S7**. Site frequency spectra (SFS) for analyses of historical demography based on all SNP markers. The best-fit demographic model identified was isolation-with-migration with exponential population growth. Shown in the upper panel are the observed (left) and expected (predicted model outcome; right) SFS. In the lower panel are the residuals from comparisons of observed and expected SFS distributions (left) and a histogram showing the distribution of those residuals. Nclade and Sclade corresponded to the northern and southern lineages of *M. californicus*. The 2D-SFS was extrapolated to a sample size of 50 alleles for each lineage.

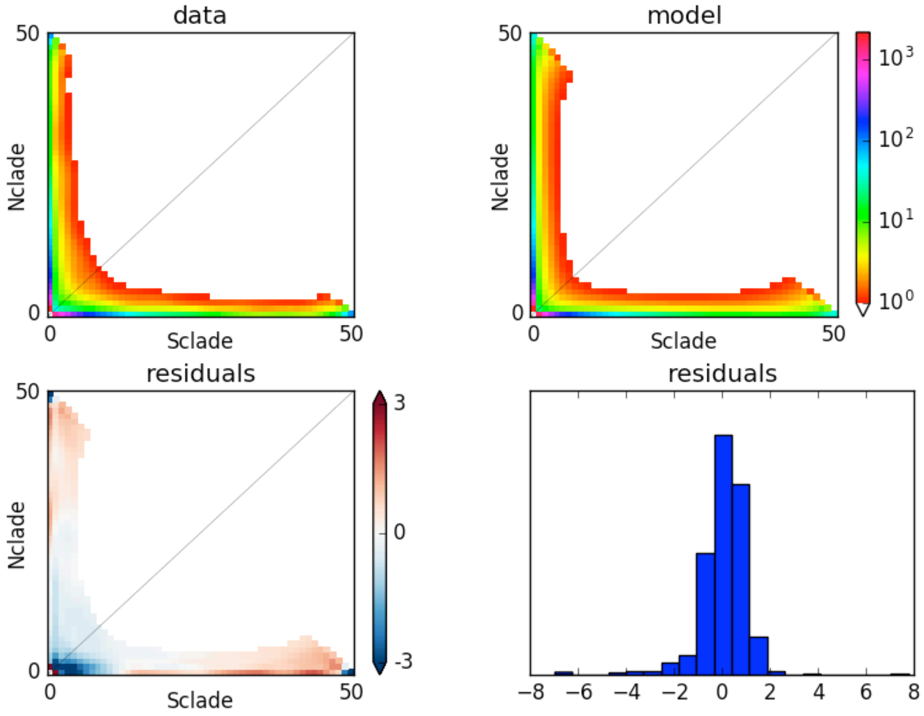

**Figure S8.** The average genomic clines estimated using relaxed (allele frequencies ≤ 0.2 and ≥ 0.8) and strict (fixed, allele frequencies = 0.0 / 1.0) cut-offs for defining diagnostic SNPs. The *cyt-b* cline is shown for comparison. See also Table S7.

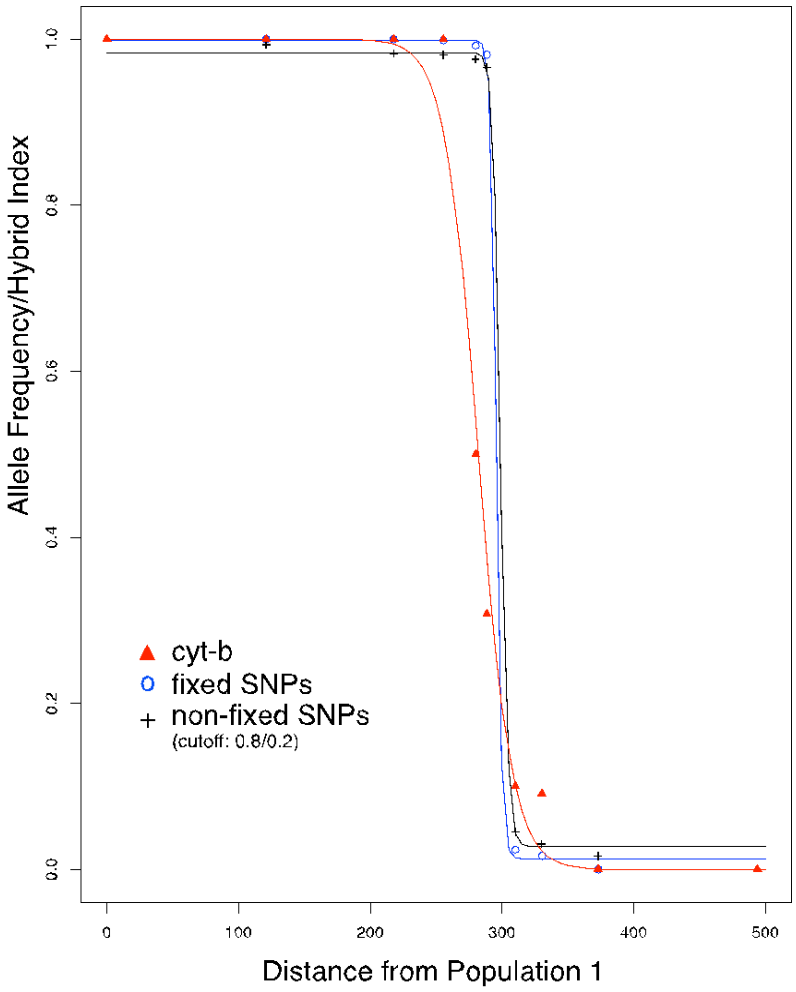

## References


Bi K, Linderoth T, Vanderpool D, *et al.* (2013) Unlocking the vault: next-generation museum population genomics. *Mol Ecol* **22**, 6018-6032.

Bolger AM, Lohse M, Usadel B (2014) Trimmomatic: a flexible trimmer for Illumina sequence data. *Bioinformatics* **30**, 2114-2120.

Coffman AJ, Hsieh PH, Gravel S, Gutenkunst RN (2016) Computationally efficient composite likelihood statistics for demographic inference. *Mol Biol Evol* **33**, 591-593.

Conroy CJ, Cook JA (2000) Molecular Systematics of a Holarctic Rodent (*Microtus*: Muridae). *Journal of Mammalogy* **81**, 344-359.

Darriba D, Taboada GL, Doallo R, Posada D (2012) jModelTest 2: more models, new heuristics and parallel computing. *Nat Methods* **9**, 772.

Fink S, Fischer MC, Excoffier L, Heckel G (2010) Genomic scans support repetitive continental colonization events during the rapid radiation of voles (Rodentia: *Microtus*): the utility of AFLPs versus mitochondrial and nuclear sequence markers. *Syst Biol* **59**, 548-572.

Guindon S, Gascuel O (2003) A simple, fast and accurate method to estimate large phylogenies by maximum-likelihood. *Syst Biol* **52**, 696-704.

Gutenkunst RN, Hernandez RD, Williamson SH, Bustamante CD (2009) Inferring the joint demographic history of multiple populations from multidimensional SNP frequency data. *Plos Genetics* **5**, e1000695.

Jaarola M, Martinkova N, Gunduz I, *et al.* (2004) Molecular phylogeny of the speciose vole genus *Microtus* (Arvicolinae, Rodentia) inferred from mitochondrial DNA sequences. *Mol Phylogenet Evol* **33**, 647-663.

Jiang H, Lei R, Ding SW, Zhu S (2014) Skewer: a fast and accurate adapter trimmer for next-generation sequencing paired-end reads. *BMC Bioinformatics* **15**, 182.

Langmead B, Salzberg SL (2012) Fast gapped-read alignment with Bowtie 2. *Nat Methods* **9**, 357-359.

Li H, Handsaker B, Wysoker A, *et al.* (2009) The Sequence Alignment/Map format and SAMtools. *Bioinformatics* **25**, 2078-2079.

Magoc T, Salzberg SL (2011) FLASH: fast length adjustment of short reads to improve genome assemblies. *Bioinformatics* **27**, 2957-2963.

McKenna A, Hanna M, Banks E, *et al.* (2010) The Genome Analysis Toolkit: a MapReduce framework for analyzing next-generation DNA sequencing data. *Genome Res* **20**, 1297-1303.

Miller MA, Pfeiffer W, Schwartz T (2010) Creating the CIPRES Science Gateway for inference of large phylogenetic trees, 1-8.

Pauperio J, Herman JS, Melo-Ferreira J, *et al.* (2012) Cryptic speciation in the field vole: a multilocus approach confirms three highly divergent lineages in Eurasia. *Mol Ecol* **21**, 6015-6032.

Peterson BK, Weber JN, Kay EH, Fisher HS, Hoekstra HE (2012) Double digest RADseq: an inexpensive method for de novo SNP discovery and genotyping in model and non-model species. *PLoS One* **7**, e37135.

Sikes RS, Mammal ACUCAS (2016) 2016 Guidelines of the American Society of Mammalogists for the use of wild mammals in research and education. *Journal of Mammalogy* **97**, 663-688.
